# Public health concern behind the exposure to persistent organic pollutants and the risk of metabolic diseases

**DOI:** 10.1186/1471-2458-12-298

**Published:** 2012-04-20

**Authors:** Jérôme Ruzzin

**Affiliations:** 1 Department of Biology, University of Bergen, Postboks 7803, 5020, Bergen, Norway

## Abstract

**Background:**

Persistent organic pollutants (POPs) are hazardous chemicals omnipresent in our food chain, which have been internationally regulated to ensure public health. Initially described for their potency to affect reproduction and promote cancer, recent studies have highlighted an unexpected implication of POPs in the development of metabolic diseases like type 2 diabetes and obesity. Based on this novel knowledge, this article aims at stimulating discussion and evaluating the effectiveness of current POP legislation to protect humans against the risk of metabolic diseases. Furthermore, the regulation of POPs in animal food products in the European Union (EU) is addressed, with a special focus on marine food since it may represent a major source of POP exposure to humans.

**Discussion:**

There is mounting scientific evidence showing that current POP risk assessment and regulation cannot effectively protect humans against metabolic disorders. Better regulatory control of POPs in dietary products should be of high public health priority.

**Summary:**

The general population is exposed to sufficient POPs, both in term of concentration and diversity, to induce metabolic disorders. This situation should attract the greatest attention from the public health and governmental authorities.

## Background

The incidence of obesity and diabetes has dramatically increased worldwide. From 1980 to 2008, the global prevalence of obesity has doubled in both men and women [1], whereas the number of people with diabetes increased from 153 million in 1980 to 347 million in 2008 [2]. A recent report realised in the European Union (EU) highlights that overweight and obesity affect more than 50% of the adult population whereas diabetes is now affecting over 30 million people [3]. In the United States (US), the prevalence of obesity among children and adolescents has almost tripled since 1980 [4], and 12% of children aged 2 through 5 years were obese in 2009-2010 [5]. In addition to enhancing the risk of premature death, diabetes and obesity are major causes of multiple complications, including hypertension, cardiovascular diseases, asthma, blindness, limb amputation, and sleep apnea, that generate enormous economic costs for both health care and loss of productivity to society. Total annual economic cost of diabetes in the US reached $132 billion in 2002, representing around 11% of the US health care expenditure, and increased to $174 billion in 2007 [6,7]. In Europe, obesity-related healthcare was estimated to reach up to 10.4 billion Euros [8].

Although they are the focus of intense investigations, the origins of metabolic diseases have remained poorly understood. While there is certainly genetic predisposition, the rapid and explosive incidence of obesity and type 2 diabetes cannot be due to genetic modifications in the population. Indeed, the propagation of these diseases is simply too fast to lie at the feet of genetic changes. Rather, environmental factors like physical inactivity and consumption of high-energy diets are likely to be important contributors. However, the view that these two conventional risk factors alone are the primary variables explaining the epidemic of metabolic diseases is being challenged as far too simplistic [9-12].

Persistent organic pollutants (POPs), including dioxins, furans, polychlorinated biphenyls (PCBs), and organochlorine pesticides, are chemicals mainly created by industrial activities, either intentionally or as by-products [13]. Because of their ability to resist environmental degradation, these substances are omnipresent in food products, and found all around the world, even in areas where they have never been used like Antarctica [14]. Thus, virtually all humans are daily exposed to POPs. In the general population, exposure to POPs comes primarily from the consumption of animal fat like fatty fish, meat and milk products; the highest POP concentrations being commonly found in fatty fish [15-26].

POPs are lipophilic chemicals that can pass through biological phospholipid membranes and bio-accumulate in fatty rich tissues of humans. Initially described for their deleterious effects on reproductive function and carcinogenicity, there is now growing body of evidence showing that exposure to POPs leads to metabolic diseases. First, many epidemiological studies performed in the US, Europe and Asia have led to the common finding that there is an increased body burden of POPs in people with diabetes [27-39]. For instance, diabetes has been associated with elevated serum levels of 2,3,7,8-tetrachlorodibenzodioxin (TCDD), 2,2',4,4',5,5'-hexachlorobiphenyl (CB153), coplanar polychlorinated biphenyls (PCBs; CB77, CB81, CB126, CB169), p,p'-diphenyldichloroethene (DDE), oxychlordane and *trans*-nonachlor. In addition, bio-accumulation of PCBs has been linked to non-alcoholic fatty liver disease (NAFLD) and elevated blood pressure [40,41]. Recently, prospective studies have furthermore shown an increased risk of diabetes in persons exposed to POPs, especially organochlorine pesticides [39,42,43]. Second, there is evidence for a causal relationship between POP exposure and metabolic disorders linked to insulin resistance. Animals exposed to environmental levels of POP mixtures through the intake of non-decontaminated fish oil (obtained from farmed Atlantic salmon) exhibited insulin resistance, glucose intolerance, abdominal obesity and NAFLD [44]. In rats fed decontaminated crude salmon oil, which contained very low levels of POPs, these metabolic disturbances were almost absent. Importantly, the body burden of POPs in rats exposed to non-decontaminated salmon oil did not exceed those observed in humans 40-50 years of age, thereby underlying that the animals were not over-exposed to POPs [44]. Further, the presence of POPs in farmed Atlantic salmon fillet was found to accelerate the development of visceral obesity and insulin resistance in mice [45]. Other studies exposing rodent to single POP compound reported metabolic dysfunctions like NAFLD or increased body weight gain [46,47]. Third, *in vitro* investigations showed that POPs are powerful disruptors of metabolic homeostasis [44-46,48], which is consistent with the obesogen properties of some xenobiotics [49]. Taken together, these findings observed in humans, animals, and cell models, demonstrate that POPs are potent environmental contributors to metabolic diseases. Based on this novel knowledge, the present article aims to stimulate discussion and to evaluate the effectiveness of current POP legislation to protect humans against the risk of metabolic diseases.

## Discussion

### Current regulation and risk assessment of POPs

To protect humans, the United Nations Environment Programme (UNEP), in 1997, issued an international action to reduce the global release of 12 POPs, the “dirty dozen”, which included nine organochlorinated pesticides; aldrin, chlordane, dichlorodiphenyltrichloroethane (DDT), dieldrin, endrin, heptachlor, hexachlorobenzene, mirex, toxaphene, and three industrial chemicals; dioxins, furans, and PCBs. In 2001, the Stockholm Convention on POPs was ratified by 151 signatories, and the Convention entered into force three years later [50].

Aldrin, chlordane, DDT, dieldrin, endrin, heptachlor, mirex, and toxaphene were extensively used in agriculture as insecticides, whereas hexachlorobenzene was used as a fungicide. By 1990, these POPs were prohibited for use on crops and termite control by most European and North American countries because of their potential threat to human health [51]. DDT has also been utilized in indoor spraying for control of vectors of malaria, dengue, visceral leishmaniasis, and Chagas disease, and its use for the purpose of controlling disease vectors has been permitted by the Stockholm Convention, in accordance with recommendations and guidelines of the World Health Organization (WHO) [52]. In its Position Statement, WHO stipulates that a sudden ban on DDT use could have significant consequences for the burden of malaria because of the absence of equally effective and efficient alternatives [52]. The use of DDT in malaria prevention has led to intense debates because it can save many lives but DDT may, at the same time, induce adverse human health effects [53-55]. In 2005, the global production of DDT used for disease vector control was estimated to reach 5,000 metric tons [56]. Up to 160 metric tons of DDT were still produced for agriculture purposes [56], and the production of other organochlorine pesticides, like aldrin and dieldrin, is suspected to occur in different countries [51]. Not surprisingly, a recent US monitoring study revealed that DDT and its metabolites as well as endosulfan and aldrin, are still largely present in food, and daily consumed by humans [19].

The term “dioxins” is commonly used to refer a group of 75 polychlorinated dibenzo-p-dioxins (PCDDs) and 135 polychlorinated dibenzofurans (PCDFs) congeners among which less than 20 are believed to be toxic. Dioxin like-PCBs (dl-PCBs) refer to 12 non-*ortho* or mono-*ortho* PCBs exhibiting similar biological pattern to dioxins. In 1997, an expert committee, set up by the WHO, developed the concept of toxic equivalent factors (TEF) to facilitate risk assessment and regulatory control of exposure to mixtures of PCDDs, PCDFs, and dl-PCBs [57]. TEF is based on *in vitro* and *in vivo* data showing that PCDD, PCDF and dl-PCB congeners induce similar toxic responses to those caused by TCDD, which is considered as the most potent toxic congener [57,58]. Thus, TEF is an order of magnitude estimate of the toxicity of a compound relative to TCDD. The criterion to apply a TEF to a POP congener is the following:

1) The chemical must show a structural relationship to PCDDs and PCDFs.

2) The chemical must bind the aryl hydrocarbon receptor (AhR) and elicit AhR-mediated biochemical and toxic responses.

3) The chemical must be ubiquitous in the food chain.

To further estimate the toxicity of POP mixtures to which humans and organisms can be exposed, TEF values are used to calculate toxic equivalent (TEQ) concentrations as follows: TEQ = Σ_n1_ (PCDDs_i_ × TEF_i_) + Σ_n2_ (PCDFs_i_ × TEF_i_) + Σ_n3_ (dl-PCBs_i_ × TEF_i_). This approach assumes that the combined effects of different PCDD, PCDF and dl-PCB congeners produce additive toxic effects.

The Stockholm Convention has led to substantial reduction of POP production. Dietary surveys have reported that the concentrations of organochlorine pesticides, dioxins, and PCBs have declined in most food products [20,59,60]. Furthermore, many studies characterizing the temporal trends of these classical POPs in human blood showed that the concentrations of these pollutants have decreased during the last years [61-64], although the human body burden of so-called “emerging” POPs, like polybrominated diphenyl ether (PBDE) and some perfluorinated compounds, have remained stable or even increased [65-67]. The present situation may therefore indicate that the current background exposure to POPs is still sufficient to perturb hormonal and metabolic homeostasis of the human body.

In general, safety guidelines have been performed by identifying the most sensitive effects of single POP, and has mainly focused on TCDD and its impacts on immunotoxicity, carcinogenotoxicity, reproductive and developmental toxicity in animals. In 2000, the Scientific Committee on Food (SCF) of the European Commission reviewed extensive data and experimental results and recommended a tolerable weekly intake (TWI) of 7 pg WHO-TEQ/kg body weight to protect consumers from the harmful effects associated with environmental contaminant exposure through consumption of food products [68]. Only six months later, the SCF re-assessed the TWI by focusing on non-carcinogenetic endpoints only [69], and increased the TWI from 7 to 14 pg WHO-TEQ/kg body weight based on rat studies investigating the reproductive effects of male offspring [70]. Below this level, contaminants are expected to have no effects or its effects are reversed by body’s defence mechanisms. Today, various authorities have defined their own maximum daily exposure limits, tolerable daily intake (TDI), within the range of 1 to 4 pg WHO-TEQ/kg body weight/day. Still, although some population has TDI below national ranges, many studies have documented that exposure to dioxins and dl-PCBs to humans, especially children, can be above the recommended TDI levels [15,21,23,24,59,71,72].

### Main limitations of current risk assessment on POPs in relation with metabolic disorders

**Figure 1:**
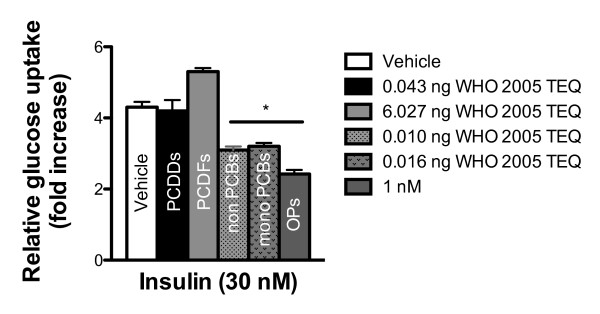
**Effect of POP mixtures on insulin action**. Differentiated 3T3-L1 adipoctytes were incubated for 48 h with different POP mixtures mimicking those present in the oil of farmed Atlantic salmon. The ability of insulin (30nM) to stimulate glucose uptake was assessed. Note that the PCDF mixture and its elevated TEQ value did not impair insulin-stimulated glucose uptake. At the opposite, PCB mixtures and their low TEQ values significantly impaired insulin action. The most important inhibitory effect on insulin action was observed after exposure to organochlorine pesticides (1nM). PCDDs, polychlorinated dibenzo-p-dioxins; PCDFs, polychlorinated dibenzofurans; PCBs, polychlorinated biphenyls; OPs, organochlorine pesticides. Data are expressed as relative glucose uptake and presented as mean ± SEM. *p < 0.05; Data are adapted from [44].

1) TEQ and the concept of additive toxic effects. Although TEQ concept is based on additive effects of pollutants, i.e. high doses of a chemical are expected to cause greater deleterious effects than low doses, not all studies on environmental pollutants and xenobiotics support this concept [42,73-77]. Accumulating data continue to underline the existence of a non-linear or inverted relationship between environmental pollutants and their detrimental effects; strong biological effects in low dose of exposure but weak or no effects in higher dose. For instance, exposure to low concentrations of TCDD and 1,2,3,4,7,8-hexachlorodibenzo-*p*-dioxin (HxCDD) increased body weight gain in rats whereas at higher doses of exposure, both TCDD and HxCDD decreased body weight [46]. Consistently, low doses of CB-77 and TCDD were found to promote adipocyte differentiation whereas higher doses resulted in the absence of cell differentiation [48]. Furthermore, 3T3-L1 adipocytes incubated with low concentration of CB-77 stimulated adipokine release whereas this effect was absent when cells were exposed to high CB-77 concentration. Overall, the assumption that the combined effects of different congeners are dose-additive, which is the most important prerequisite of the TEQ concept, is highly questionable and linear extrapolation procedure is likely to under-estimate the metabolic risks linked to POP exposure.

2) TEF, TEQ and the assumption that dioxins and dl-PCBs exhibit similar types of biological effects through interaction with AhR. TEF concept assumes that, among POPs, TCDD is the most toxic chemical. Therefore, the potency of dioxins, furans and dl-PCBs has been expressed as fractions of the potency of TCDD. Although TCDD, through AhR activation, may induce cancer, reproduction and neuronal dysfunctions, TEQ is unlikely to predict the risk of metabolic disorders induced by POP exposure. For instance, the ability of different POP mixtures to impair insulin action in 3T3L1 adipocytes was reported to be independent of TEQ (Figure 1). Both PCDD and PCDF mixtures did not impair insulin-stimulated glucose uptake compared with vehicle-treated cells, whereas non- and mono-ortho-PCB mixtures induced significant disruption of insulin action (Figure 1). Interestingly, the stimulation of glucose uptake induced by insulin was highly impaired after exposure to mixtures of organochlorine pesticides. These *in vitro* findings are consistent with many human studies indicating that POPs increase the risk of diabetes independently of TEQ, and that some organochlorine pesticides, like *trans*-nonachlor, are more strongly associated with diabetes than other POPs [29,42,78]. Consistently, *in vitro* experiments using single POP compound reported that, at similar TEQ value, CB-77 stimulated adipocyte differentiation whereas this effect was absent in cells treated with TCDD [48]. In concert, these results suggest that the deleterious metabolic effects associated with POP exposure may occur independently of AhR activation. Potential modes of action of POPs may include activation of constitutive androstane receptor (CAR) or steroid xenobiotic receptor (SXR), and competitive binding to nuclear receptors [79,80].

3) Adult and early life exposure. There is a special concern regarding children because regulatory policies, which established dioxins and dl-PCBs tolerable intake on a body weight basis, do not distinguish between children and adults. Because of their high food intake per kilogram body weight required to maintain whole-body homeostasis and growth, children are likely to be at higher risk for environmental pollutant exposure. Not surprisingly, many scientific studies have highlighted that children are over-exposed to dioxins and dl-PCBs, and exceed the TDI of 2 pg/kg body weight [15,21,23,81-87]. Furthermore, both metabolism, absorption and excretion system are different in children compared with adults, so that this subpopulation may potentially be more responsive to environmental pollutants [88]. Another important issue concerns foetal life. Generally, pregnant women have been advised to avoid the consumption of food containing elevated levels of POPs, as well as other environmental pollutants like heavy metals. However, POPs bio-accumulate in the body for many years, and restricting the exposure to these pollutants only during pregnancy would not protect the foetus or the future generations against the harmful effects of these hazardous chemicals [89].

### Inconsistency of POP regulation in animal food products: the case of marine food

The omnipresence of POPs in animal food products has led international and national authorities to set up maximum limits. In this regard, marine food has attracted great interest and has been the source of intense debates [90-92]. In the EU, maximum limit of dioxins, furans and dl-PCBs in fish and seafood products have been set to 8 pg WHO 1998 TEQ/g fresh weight [93]. Giving that these POPs are highly lipophilic and therefore mainly present in the fat fraction of fish, this concentration could reach around 70 pg WHO 1998 TEQ/g fat for a fatty fish (Table 1). On the other hand, the maximum limit of dioxins, furans and dl-PCBs in marine oil has been set to 10 pg WHO 1998 TEQ/g fat, a level that is seven times lower the one possibly observed in muscle meat of fatty fish. Thus, eating 1 g of fat from a fatty fish fillet could induce an exposure to 70 pg WHO 1998 TEQ (depending on the fat content of fish), whereas consuming 1 g of fat from a marine oil product could lead to an exposure to 10 pg WHO 1998 TEQ. Since the maximum levels for dioxins and dl-PCBs in foodstuffs should be set according to their potential harmful effects and risk to public health, one would expect these levels to be harmoniously regulated within food products.

**Table 1:** Regulation of dioxins, furans and dl-PCBs in fish and other animal food products by the EU

	**Maximum levels of dioxins, furans and dl-PCBs (WHO 1998 TEQ)**
**Meat and meat products from:**	
Ruminants:	4.5 pg/g fat
Poultry and farmed game:	4.0 pg/g fat
Pigs:	1.5 pg/g fat
**Muscle meat of fish and fishery products**^**1**^**:**	
	8.0 pg/g fresh weight (70.0 pg/g fat)^2^
**Fat products from:**	
Ruminants:	4.5 pg/g fat
Poultry and farmed game:	4.0 pg/g fat
Pigs:	1.5 pg/g fat
Marine oils:	10.0 pg/g fat

^1^except eel. ^2^assuming a fish fillet containing 12% fat like farmed Atlantic salmon.

Note that the maximum limits of POPs in ruminants, poultry and farmed game, and pigs are similar in the “**Meat and meat products from**” compared with “**Fat products from**”. In contrast, “**Muscle meat of fish and fishery products**” could contain up to 70 pg WHO 1998 TEQ/g fat, which is around seven times higher the maximum limit set in “**Fat products from marine oils**” (10 pg WHO 1998 TEQ/g fat). Adapted from [93].

Another important issue is the regulation of organochlorine pesticides, which are chemicals strongly linked to type 2 diabetes [29,32,33,37,44,45] as well as breast and prostate cancer [94] and Parkinson disease [95]. As for dioxins, furans and dl-PCBs, the EU has set up maximum residue levels in or on food and feed of plant and animal origin [96]. Thus, fruits, vegetables, products of terrestrial animal origin, cereals, and other food items are regulated for their content in pesticides. However, levels of pesticides are still unregulated in fish and seafood in the EU. Similarly, there are still no maximum limits for PCBs in marine food. Because seafood represents one of the main sources of human exposure to POPs, and that POPs contribute to metabolic diseases, policy makers and stakeholders should urgently regulate and diminish the concentrations of POPs in seafood to protect the general population. Indeed, recent studies have reported an association between fish and seafood intake and type 2 diabetes in humans [97-99], and possible connection between farmed Atlantic salmon and the epidemic of type 2 diabetes has been suggested [100].

Further, some EU countries, like Finland and Sweden, have obtained dispensations to sell fish species from the Baltic sea, a highly polluted area where fish species can accumulate significant levels of POPs -dioxin levels higher than 4 pg/g fresh weight of muscle meat and fishery products are allowed [93]. Also, the maximum levels of dioxins and dl-PCBs in muscle meat of eel, a potential highly contaminated fish, reached 12 pg WHO 1998 TEQ/g fresh weight, in contrast to the 8 pg WHO 1998 TEQ/g fresh weight of other fish species [93]. Such derogations likely rose from the fact that background contamination of certain foodstuffs is already very high -mainly because of human pollution- and that keeping maximum dioxins and dl-PCBs levels similar in all food products would result to declare considerable part of our present food supply unfit for human consumption [68]. Unfortunately, this strategy is not aimed to reduce human exposure to POPs and raise serious questions about the effectiveness of EU regulations to protect consumers.

## Summary

The global prevalence of metabolic diseases like obesity and type 2 diabetes, and its colossal economic and social costs represent a major public health issue for our societies. There is now solid evidence demonstrating the contribution of POPs, at environmental levels, to metabolic disorders. Thus, human exposure to POPs might have, for decades, been sufficient and enough to participate to the epidemics of obesity and type 2 diabetes. Based on recent studies, the fundaments of current risk assessment of POPs, like “concept of additive effects” or “dioxins and dl-PCBs induced similar biological effects through AhR”, appear unlikely to predict the risk of metabolic diseases. Furthermore, POP regulation in food products should be harmonized and re-evaluated to better protect consumers. Neglecting the novel and emerging knowledge about the link between POPs and metabolic diseases will have significant health impacts for the general population and the next generations.

## Competing interests

I have no competing interests to declare.

## Author’s information

JR has been working on environmental factors linking to obesity and type 2 diabetes. During the last 5 years, his research studies have focused on environmental pollutants, and he demonstrated, for the first time, the causal role of POPs in the development of metabolic disorders. Recently, part his work was presented at the High Level Consultative Conference of the Environment and Public Health Community in Europe, Berlin-Potsdam, Germany.

## Pre-publication history

The pre-publication history for this paper can be accessed here:

http://www.biomedcentral.com/1471-2458/12/298/prepub
